# Evaluation of the implementation of a mobility plan in a geriatric clinic in Switzerland – a quality improvement study

**DOI:** 10.1186/s12877-026-07664-8

**Published:** 2026-05-23

**Authors:** Victoria Eva Schäppi-Dändliker, Ylena Fuchsberger, Johannes Pohl, Dominik Kunz, Pierrette Baschung Pfister

**Affiliations:** 1 School of Health Sciences, ZHAW Zurich University of Applied Sciences, Winterthur, Switzerland; 2 University Centre for Prevention and Sports Medicine, Balgrist University Hospital, Zurich, Switzerland; 3 Physiotherapy Occupational Therapy, Nursing and Allied Health Profession Office, University Hospital Zurich, Zurich, Switzerland; 4 Data Analytics & Rehabilitation Technology (DART), Lake Lucerne Institute, Vitznau, Switzerland; 5 Research Centre for Computational Health, Institute of Computational Life Sciences, ZHAW Zurich University of Applied Sciences, Wädenswil, Switzerland; 6 Directorate of Research and Education, Physiotherapy Occupational Therapy Research Centre, University Hospital Zurich, Zurich, Switzerland

**Keywords:** Implementation Science, Quality improvement, Mobility Plan, Steps, Motor Activity, Geriatrics, Inpatients, Activities of Daily Living, Hospitalisation, Aged

## Abstract

**Background:**

Patient inactivity during hospitalisation is a widespread issue, particularly affecting older adults and contributing to functional decline and prolonged hospital stays. Mobility Plans are a promising strategy to support goal-directed mobilisation, yet their implementation remains challenging. This quality improvement study aimed to evaluate the success of implementing the Mobility Plan in a Swiss geriatric clinic. The secondary objective was to describe inpatient mobility before and during implementation.

**Methods:**

A within-site quality improvement design was used, guided by the Implementation of Change Model and the Implementation Research Logic Model. Five implementation outcomes - acceptability, appropriateness, feasibility, fidelity, and adoption - were evaluated through healthcare professional questionnaires and analysis of the Mobility Plan documentation. Inpatient mobility was assessed using step counters in two independent four-week evaluation phases: one before and one during the three-month implementation of the Mobility Plan. Descriptive statistics were applied to all outcomes.

**Results:**

Therapists reported agreement rates above the predefined 50% threshold for all implementation outcomes at the subscale level (acceptability: 68.2%, appropriateness: 90.9%, feasibility: 90.9%), whereas nurses reached the threshold only for appropriateness (56.7%) but not for acceptability (23.3%) or feasibility (45%). No physicians responded to the questionnaire. A total of 69 Mobility Plans were analysed. Fidelity, defined as the proportion of days with a documented mobility objective, reached a median of 75% [IQR 56–86%], while adoption, defined as the proportion of days with at least one recorded mobility activity, reached 88% [IQR 80–92%]. Median daily step counts were descriptively higher on all four measurement days in the second evaluation phase compared with the first.

**Conclusion:**

The implementation of the Mobility Plan showed mixed results. It appeared feasible to apply in daily practice, although engagement varied across healthcare professionals. A small increase in daily steps was observed during the implementation phase, although this cannot be linked directly to the Mobility Plan because of the study design. Further work is needed to strengthen interdisciplinary uptake and to examine its potential effects on patient mobility using more robust study designs.

**Trial registration:**

Clinical trial number: not applicable.

**Supplementary Information:**

The online version contains supplementary material available at 10.1186/s12877-026-07664-8.

## Background

### Problem statement and relevance

Patient mobility in hospitals is a key factor for recovery and the preservation of functional independence. However, studies have shown that most inpatients spend 87–100% of their time lying or sitting, often taking fewer than 1000 steps per day [1–4]. This high level of inactivity is associated with negative outcomes, such as functional decline, prolonged hospital stays, and increased healthcare costs [3–5]. In older adults in particular, reduced mobility during hospitalisation has been linked to an increased risk of delirium, falls and long-term dependency [4, 5]. Interventions such as the “Hospital in Motion” program demonstrate that structured, multidimensional approaches can effectively improve patient movement behaviour during hospital stays [6].

### Previous findings and knowledge gap

To address the physical inactivity among inpatients various multidimensional interventions have been developed, such as mobility cards, QR-code-based walking routes, joint meals, and resistance training [6]. However, a Swiss study of older inpatients revealed that 9.1% of their daily activity was of only light intensity, and just 0.4% was classified as moderate [7]. This highlights that implementing mobility-promoting strategies in everyday hospital practice remains difficult. Another approach to stimulate physical activity in inpatients is the Mobility Plan (MP), which is based on the Johns Hopkins Highest Level of Mobility Scale and supports the visualization of daily mobility goals and progress tracking [8, 9]. Both aspects – goal setting and feedback by tracking progress – are indicated as enhancing mobility during hospitalisation [8, 10, 11]. The geriatric clinic of the University Hospital Zurich attempted to use the MP previously but encountered several barriers, such as no therapy planned on weekends, insufficient integration into ward rounds, and limited familiarity with the MP among nursing staff.

### Objectives

To overcome these barriers, a targeted implementation strategy was developed in 2023. To develop this strategy in a structured and theory-informed manner, two established frameworks were followed: the Implementation of Change Model by Grol et al. [12] and the Implementation Research Logic Model by Smith et al. [13]. These models supported the identification of relevant contextual factors and the selection of tailored implementation strategies. This study aimed to evaluate the implementation success of the MP based on healthcare professionals’ (HCP) agreement rates (acceptability, appropriateness, feasibility) and documentation metrics (fidelity, adoption). A simple explanation of these terms can be found in Table 1 in the method section. From the patient perspective, mobility was described by quantifying step counts before and during implementation.

## Methods

### Study design

This quality improvement study used a within-site design, and the SQUIRE guidelines were followed in conducting the study [14]. Within-site design refers to a quasi-experimental approach in which data are collected within a single setting before and after the implementation of an intervention. This approach made it possible to examine changes over time within the same clinical setting. It is particularly useful when randomization is not feasible, and it supports the evaluation of implementation outcomes in real-world contexts [15]. In this manuscript, the term MP refers to the goal-setting tool, whereas intervention refers to the broader implementation strategy supporting its use.

### Setting

This study was conducted at the geriatric clinic of the University Hospital Zurich from April to August 2023 in Switzerland.

### Study sample

**Fig. 1:**
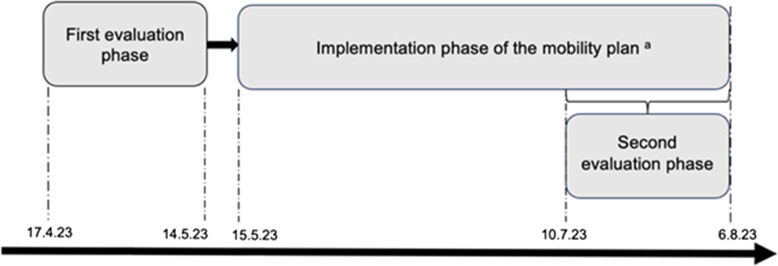
Implementation and evaluation timeline ^a^ Responsability of the geriatric clinic

The sample for evaluating the implementation success comprised all HCPs, including physicians, nurses, and therapists working at the geriatric clinic during the implementation phase of the MP. Furthermore, all MPs from the entire implementation phase were utilized to assess the daily documentation and objectives. Figure 1 illustrates the procedure. The sample considered for the secondary goal – measuring the mobility by accelerometers – included all inpatients who were present at the geriatric clinic during the corresponding evaluation phase. The subject selection criteria included proficiency in German language skills, providing written informed consent and a minimum length of stay of at least seven days. The exclusion criteria included a Mini-Mental State Examination Score < 20 (indicating mild cognitive impairment) [16], bed rest, and preexisting immobility before hospital admission. The accelerometer was utilized in individuals who expressed a willingness to wear it and met the inclusion criteria. The aim was to measure twelve inpatients per evaluation phase, each wearing the accelerometer for four consecutive days. The sample size was determined based on the specific setting and considered feasible for the chosen measurement period.

### Implementation outcomes

To evaluate the success of the MP implementation, five specific implementation outcomes were defined, based on the framework by Proctor et al. [17]: appropriateness, acceptability, feasibility, fidelity, and adoption. These outcomes were selected as essential preconditions for successful and sustainable improvements in patient-level outcomes. To clarify the specific terms used to describe implementation outcomes, Table 1 provides a concise overview with simple definitions.

**Table 1:** Selected implementation outcomes

	Appropriateness	Acceptability	Feasibility	Fidelity	Adoption
Definition^a^	Perceived fit of the intervention to address a particular problem	Perception among stakeholders that the given intervention is agreeable	Extent of successful application of the intervention in the setting	Degree to which the intervention was implemented as it was intended by the developers	Intention to employ the intervention

^a^The Mobility Plan serves as the intervention in this context

Definitions adapted from Proctor et al. [17]

Acceptability, appropriateness, and feasibility were measured using a German-language questionnaire comprising 12 items (four per construct). We used an existing German translation of the Acceptability of Intervention Measure, Intervention Appropriateness Measure and Feasibility of Intervention Measure questionnaire. Accordingly, no forward–backward translation procedure or pretesting was performed within this study. Psychometric evaluation of this German version has demonstrated a three-factor structure consistent with the original instruments, high internal consistency (Cronbach’s α = 0.91–0.97), and evidence of construct validity [18]. Each item was rated on a five-point Likert scale (‘completely’ disagree to ‘completely agree’). For more details, see the full German version in Appendix 4. A target threshold of ≥ 50% agreement was defined per implementation outcome, reflecting a minimally sufficient level of stakeholder endorsement. In line with the pragmatic quality-improvement context and the small and heterogeneous sample, the three implementation outcomes were analysed at the item level and as composite scale scores. For each item, the proportion of respondents selecting ‘agree’ or ‘completely agree’ was calculated and reported as the agreement rate.

Adoption and fidelity were operationalised based on clinical documentation and analysed descriptively, reported as medians with interquartile ranges.

Fidelity was operationalised as the proportion of hospitalisation days on which a mobility objective was documented. Adoption was defined as the proportion of days with at least one recorded mobility activity. In the absence of validated thresholds for documentation-based implementation indicators in geriatric inpatient settings, ≥ 70% (fidelity) and ≥ 80% (adoption) were applied as pragmatic benchmarks, reflecting the extent to which the MP was documented in routine clinical practice. This approach is aligned with recommendations for pragmatic measurement in implementation research [19] and real-world quality-improvement evaluation [20].

These differentiated thresholds reflect the diverse nature of implementation outcomes: perceptual measures such as acceptability may require moderate levels of agreement to support MP continuation, whereas behavioural outcomes like fidelity and adoption require higher thresholds to ensure integration and sustained impact.

Engagement was not measured as a separate outcome but inferred from questionnaire response rates and agreement levels on acceptability, appropriateness, and feasibility.

### Client outcome

To evaluate the inpatient mobility before and at the end of the implementation phase, function – defined as one of the client outcomes in Proctor et al.’s framework and representing a patient-level outcome in this study – was selected as the outcome measure [17]. In this study, the client outcome function was operationalised as inpatient mobility and was objectively assessed using the GENEActiv accelerometer, which recorded daily step counts. The GENEActiv has demonstrated itself as a reliable and valid measuring instrument for quantifying step counts [21]. To prevent potential skin irritation from attaching the tracker to the ankle, the accelerometer can be worn over socks or bandages. The raw accelerometer signals were processed using a custom step counter method to detect signal peaks. Previous studies suggested the StepWatch activity monitor (SAM) as a suitable standard to assess step counts in a clinical setting [21]. Therefore, data samples from two inpatients were collected before the start of the first evaluation phase, and the SAM and GENEActiv sensors were used simultaneously to calibrate the parameters of the custom step counter. The difference in step counts from the SAM resulted in a mean absolute percentage error of 2.5%, which was deemed acceptable for this task. The daily step counts were recorded with the accelerometer from the third to the end of the sixth day of hospitalisation in both evaluation phases. Accelerometer measurements were conducted over four consecutive days without distinguishing between specific weekdays or weekend days. Furthermore, each of the included inpatients received an information sheet about the correct use of the accelerometer (complete form in Appendix 2). The use of the accelerometer was complemented with an anonymous patient questionnaire (complete form in Appendix 3) to gather age, walking aid and physical activity prior to hospital admission. Physical activity level was categorized into three levels, based on the reported amount of time spent performing activities of moderate or high intensity. Moderate intensity was defined as activity causing slight breathlessness, while high intensity referred to activities causing significant breathlessness or involving active sport [22].

### Study procedure

In 2023, the first evaluation phase from April 17 to May 14 occurred directly before the start of the implementation phase of the MP. The second evaluation phase, from July 10 to August 6, took place at the end of its implementation (more details about the timeline are provided in Fig. 1). It is important to note that evaluation phase 1 and evaluation phase 2 comprised independent samples of hospitalised geriatric patients. Due to the fluctuating inpatient population and short lengths of stay, a repeated-measures design was not feasible in routine care. Therefore, a pre–post comparison with independent samples was applied as a pragmatic approach in this quality improvement context. Each phase involved a distinct inpatient sample, recruited during separate four-week periods before and during the implementation of the MP. Prior to the two evaluation phases, the evaluation manager briefed all HCPs during the morning report about the purpose and procedure of the study and their role in it. After this briefing, the relevant information was displayed on the electronic screen at the nurses’ station. This approach aimed to keep all HCPs informed, including those who might have missed the briefing. In addition, training sessions were conducted for those team members who were executing the step measurements. Prior to the implementation phase, the staff of the geriatric clinic formed a working group with champions from various professions. The goal was to share the current project status and understand all perspectives of the included HCPs regarding implementation processes. In developing the implementation strategy, insights from interviews with HCPs on individual barriers of the MP to implementing the MP were combined with guidance from two theoretical frameworks to address both practical and conceptual aspects. Afterward, the infrastructure was adapted, and the materials were adjusted as necessary (e.g., displaying the MP and a reminder bar in the clinic documentation system). Training sessions with all HCPs were then conducted, and materials were distributed.

### First evaluation phase

Two therapists working in the geriatric clinic were responsible for the preselection of potential inpatients for the step measurements on their first hospitalisation day. In the following days, rotating four therapists provided oral information about the study, obtained additional information from inpatients, screened for exclusion, correctly placed the accelerometer and collected the used ones with the corresponding patient questionnaire. For further details, see the flowchart of inpatient recruitment and measurement procedures in Appendix 1.

### Implementation phase

**Fig. 2:**
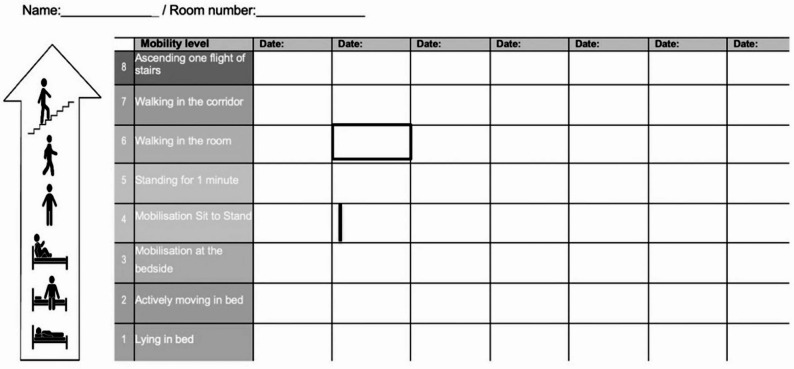
Mobility plan

The staff of the geriatric clinic implemented the MP with the aim of instructing all inpatients to stay more active by using it. Considering the implementation strategy, regular reminders and training on the MP were provided to the HCP during the implementation phase. The MP consists of an 8-point ordinal scale ranging from lying in bed (activity level 1) to ascending one flight of stairs (activity level 8). While nurses documented the daily mobility objective in the MP, the goals themselves were jointly defined in collaboration with therapists and in agreement with the patient, based on the patient’s functional status, mobility potential, and therapy recommendations. At the beginning of the day, the nurse documented the daily mobility objective for each day on the plan by outlining the corresponding field (e.g., see Fig. 2 at mobility level six). Throughout the day, every mobility performed should be recorded by lines in the field of the activity conducted by the nurses, therapists and the inpatients of the geriatric clinic (e.g., see Fig. 2 at mobility level four). Nurses collected all the MPs at hospital discharge.

### Second evaluation phase

The procedures for the step measurements were equivalent to those of the first evaluation phase. In the second evaluation phase, the German translation of the Acceptability of Intervention Measure, the Intervention Appropriateness Measure and the Feasibility of Intervention Measure was used to assess implementation outcomes. The questionnaire was distributed via email and in print to all HCPs working during the implementation phase in the geriatric clinic.

### Data analysis

To evaluate the implementation success, descriptive statistics were used to analyse HCPs perceptions and the documentation of the MP. For each item of the questionnaire, the percentage distribution of responses across the five-point Likert scale (‘completely disagree’ to ‘completely agree’) was calculated. For each item related to acceptability, feasibility, and appropriateness, an agreement rate of at least 50% was defined as the threshold for success. Documentation-related metrics were used to assess adherence to the MP. The proportion of hospitalisation days with at least one documented mobility activity (at least one line per day) was used to quantify adoption, while the percentage of days with a predefined daily mobility objective was used to evaluate fidelity.

Patient mobility was measured using the GENEActiv accelerometer, which recorded daily step counts between 8:00 AM and 8:00 PM. Raw data were exported and processed with a custom step detection algorithm in Python (3.10.9). Data normality was evaluated through visual inspection and the Shapiro-Wilk test. Due to non-normal distributions, all continuous data are presented as medians and IQRs. Step count calculations were conducted in Python (3.10.9), and all statistical analyses were performed using RStudio.

## Results

### Fidelity and adoption of the mobility plan

In total, 69 MPs were analysed during the entire implementation phase. The median percentage of all documented hospitalisation days in the MP (with at least one line per day) was 88% [IQR 80–92%]. Moreover, the median percentage of days with a mobility objective outlined (further details in Table 2) was 75% [IQR 56–86%]. Furthermore, 87.7% of all daily goals in the MP were achieved.

**Table 2:** Fidelity and adoption of the Mobility Plan

	value (n or days)	% of total MPs
Total number of MPs (n)	69	100%
Inpatients’ length of stay (days)	Median 8 [IQR 7–11]	
MPs documented (days)	Median 7 [IQR 6–9]	Median 88% [IQR 80–92%]
Daily objectives (days)	Median 6 [IQR 4–8]	Median 75% [IQR 56–86%]

*Abbreviations*: *MPs *Mobility Plans, *IQR *Interquartile range

### Appropriateness, acceptability and feasibility of the mobility plan

**Fig. 3:**
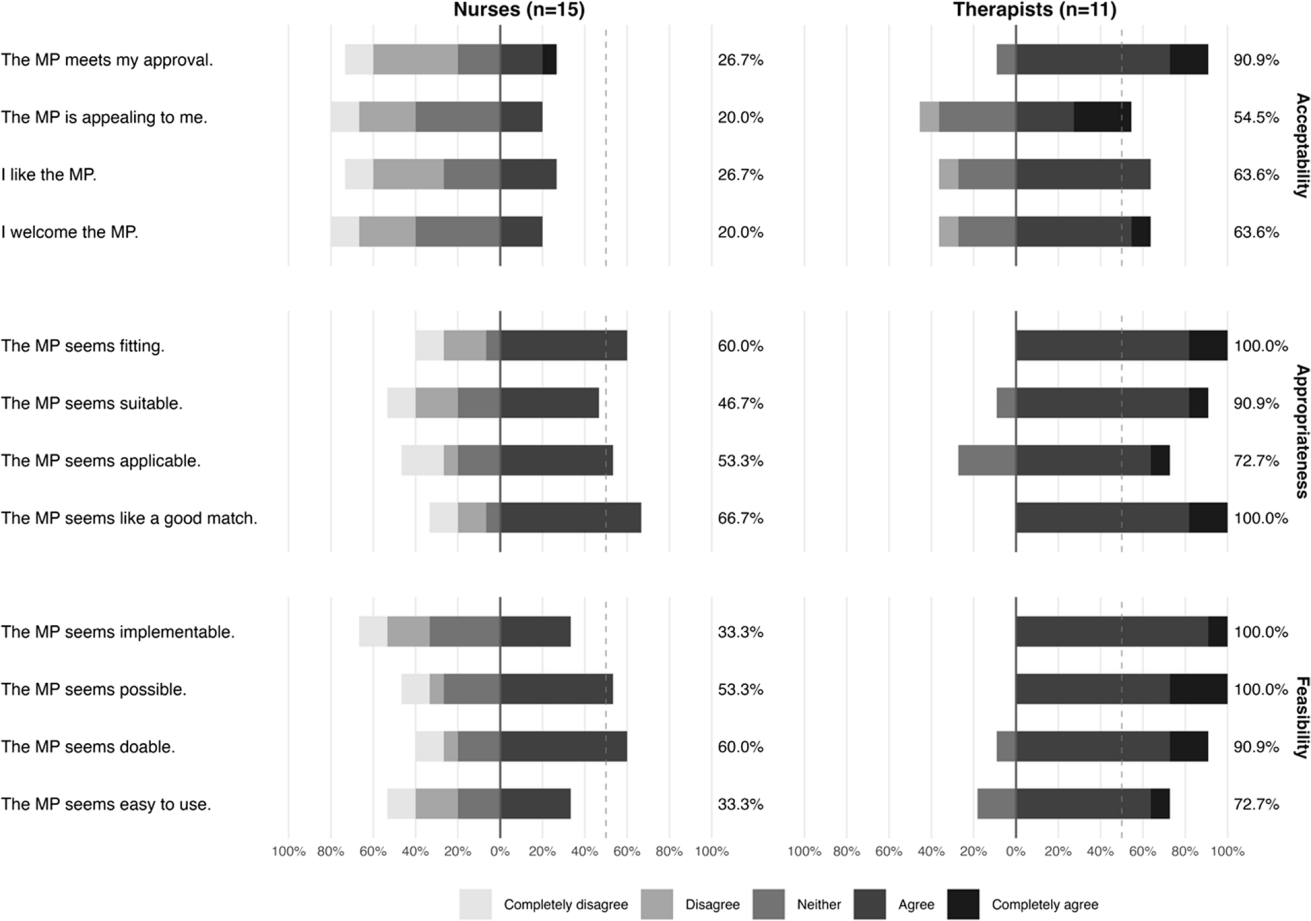
Item-level agreement with the mobility plan among nurses and therapists Abbreviation: MP, Mobility Plan The figure displays the percentage distribution of responses across the five-point Likert scale (1 = completely disagree to 5 = completely agree) for 12 items comprising three implementation outcomes: Acceptability (items 1-4), Appropriateness (items 5-8), and Feasibility (items 9-12). Shading represents response categories from 'completely disagree' (lighest grey) to 'completely agree' (darkest grey). The solid vertical line at 0% separates neutral and negative responses (left) from positive responses (right). The dashed vertical line at 50% indicates the predefined threshold for successful implementation. Percentages to the right of each bar show the proportion of respondents who selected 'agree' or 'completely agree' for that item. For statistical summaries including median Likert scores and interquartile ranges, see Table 4. Response rates: Nurses 30% (*n*=15/50); Therapists 73% (*n*=11/15); Physicians 0% (*n*=0/3). Interpretation of between-profession differences should be made with caution due to unequal sample sizes and the absence of physician responses

As shown in Table 3, out of a total of 68 HCPs working in the geriatric clinic, 15 nurses (total: 50), 11 therapists (total: 15) and no physicians (total: 3) responded to the questionnaire to evaluate the success of the implementation of the MP. At the item level, all four appropriateness items and all four feasibility items achieved >70% agreement among therapists, while among nurses, three of four appropriateness items (60.0%, 53.3%, 66.7%) and two of four feasibility items (53.3%, 60.0%) exceeded 50% agreement. Additional details are shown in Figure 3. Relative to the predefined 50% agreement threshold, therapists exceeded this criterion for all three implementation outcomes at the subscale level (acceptability: 68.2%, appropriateness: 90.9%, feasibility: 90.9%), whereas nurses reached the threshold only for appropriateness (56.7%) but not for acceptability (23.3%) or feasibility (45.0%). The median [IQR] ratings and the proportion of HCPs agreeing or completely agreeing with each implementation outcome are provided in Table 4.

**Table 3:** Sample of healthcare professionals

	Total (n)	Response to the questionnaire ^a^ [% (n)]
Physicians	3	0% (0/3)
Nurses	50	30% (15/50)
Therapists	15	73% (11/15)
Total	68	38% (26/68)

^a^see Appendix 4 for the questionnaire

**Table 4:** Summary of acceptability, appropriateness and feasibility of the Mobility Plan by nurses and therapists

Implementation outcomes	HCPs	n	Median (IQR)	% agree / completely agree
Acceptability	Nurses	15	2.75 [2–3.25]	23.3
Acceptability	Therapists	11	3.75 [3.25–4.2]	68.2
Appropriateness	Nurses	15	3.75 [2–4]	56.7
Appropriateness	Therapists	11	4 [3.75–4]	90.9
Feasibility	Nurses	15	3 [2.5–3.75]	45
Feasibility	Therapists	11	4 [4–4.25]	90.9

*Abbreviations*: *IQR *Interquartile range, *HCPs *Healthcare professionals

Values represent medians [IQR] across items within each implementation outcome and the percentage of respondents selecting ‘agree’ or ‘completely agree’

### Mobility of inpatients

The characteristics and mobility-related indicators of inpatients wearing the accelerometer during the hospital stay are summarized in Table 5. In the first evaluation phase, 13 out of 23 inpatients (6 men, 7 women) were enrolled, whereas in the second evaluation phase, 8 of the 23 possible inpatients (3 men, 5 women) participated. In general, inpatients in phase one exhibited higher levels of physical activity prior to hospital admission compared to those in phase two. While all inpatients in phase one required a walking aid, only 5 out of 8 inpatients in phase two required such support. Regarding inpatient mobility, the median daily step count consistently exceeded that of phase one on each day of phase two (further details are provided in Table 6).

**Table 5:** Characteristics of inpatients wearing the accelerometer during hospital stay

	Evaluation phase 1(n/N = 13/23; 56.5%)	Evaluation phase 2(n/N = 8/23; 34.8%)
Age (in years)	Median 82 [IQR 79–89]	Median 88.5 [IQR 81.8–91.3]
Required a walking aid (n)	13	5
Physical activity prior to hospital admission ^d^	Level 1: 46.2% ^a^	Level 1: 62.5% ^a^
	Level 2: 23.1% ^b^	Level 2: 25% ^b^
	Level 3: 30.8% ^c^	Level 3: 12.5% ^c^

*Abbreviations*: *IQR *Interquartile range, *SD *Standard deviation

^a^Level 1 = < 2.5 hours moderate intensity or 1.25 hours high intensity

^b^Level 2 = approximately 2.5 hours of moderate intensity or 1.25 hours of high intensity

^c^Level 3 = > 2.5 hours moderate intensity or 1.25 hours high intensity

^d^Self-reported physical activity prior to hospital admission; not assessed with the accelerometer

Values in the column headers represent the number of inpatients wearing the accelerometer relative to the total eligible inpatient population (n/N) during each evaluation phase. Percentages indicate the proportion of eligible inpatients who participated in the accelerometer measurement

**Table 6:** Daily step counts during the evaluation phases

	Evaluation phase 1(n = 13)	Evaluation phase 2(n = 8)	Difference ^a^
Day 1	Median 1391[IQR 854–2545]	Median 1485[IQR 899–2812]	Median 94
Day 2	Median 1964[IQR 1368–2631]	Median 2051[IQR 1482–3192]	Median 87
Day 3	Median 2121[IQR 1656–3437]	Median 2289[IQR 1343–4357]	Median 168
Day 4	Median 1990[IQR 1672–2717]	Median 2146[IQR 1517–3043]	Median 156

*Abbreviation*: *IQR *Interquartile range

^a^Between phase 2 and phase 1

## Discussion

The implementation of the MP was partially successful, as reflected by variable engagement across HCP groups. Differences in acceptability, appropriateness, and feasibility indicate that not all HCPs adopted the MP equally. While therapists showed consistently high agreement with all items, nurses reported lower ratings for several constructs. This is consistent with previous reports highlighting that mobility promotion often competes with acute nursing priorities and is perceived as less central to nurses’ responsibilities [23]. Fidelity reached a median of 75%, and adoption was high at 88%, indicating that the MP was applied on most days, although inconsistent engagement may have affected implementation quality.

The limited response rate likely reflects workload and competing priorities [24]. Response rates differed substantially between professional groups, with higher participation among therapists (11/15; 73%) compared to nurses (15/50; 30%), and no responses from physicians. As only few nurses and no physicians responded, conclusions regarding multidisciplinary acceptability remain constrained. These circumstances limited our insight into HCP-specific attitudes and reduced the extent to which the results can be transferred to other settings. These challenges align with previous findings identifying insufficient role clarity and shared responsibility as key barriers to mobility promotion in hospital settings [25].

Contextual factors may also have influenced implementation. Towards the end of the second evaluation phase, hospital management announced the relocation and merger of the geriatric clinic, potentially undermining long-term commitment among staff. This may partially explain the low questionnaire participation among nurses and physicians.

A modest increase in daily step counts was observed following the implementation of the MP, which aligns with previous evidence showing that structured mobilisation interventions can improve mobility levels in hospitalised older adults without compromising safety [26]. Individual goal setting and progress feedback, both integrated into the MP, have also been associated with enhanced mobilisation and motivation [10, 27]. This suggests that straightforward tools like the MP may fit well into routine geriatric care and help support mobilisation. Regarding the assessment of mobility, a positive trend in daily step count was observed during the second evaluation phase. Differences in age, walking-aid use, and pre-admission physical activity between the two independent samples may have contributed to the observed differences in step counts, independent of the intervention. Daily step counts in evaluation phase 1 and phase 2 were obtained from two independent inpatient groups treated on the same acute geriatric ward at different time periods, consistent with a within-site design. Previous accelerometer studies in hospitalized older adults report typical inpatient values of 600–800 steps/day and suggest that accumulating < 900 steps/day is associated with increased risk of functional decline at discharge [28, 29]. In our study, both evaluation-phase samples exceeded this threshold substantially (median 1391–2289 steps/day), indicating comparatively higher baseline mobility. Against this background, the modest between-phase differences observed here are unlikely to represent clinically meaningful functional variation. As the functional relevance of step-count differences depends strongly on baseline mobility, small absolute differences may have limited impact in more mobile patients, whereas relative (percentage) changes may be more informative in low-mobility groups. Future studies should therefore examine step-count changes in relation to baseline mobility to better capture clinically meaningful improvement. While step count provides a valuable measure of mobility, it does not reflect movement intensity. Particularly in geriatric populations, low-intensity or minimal steps may not translate into functional mobility gains. Therefore, complementary measures such as heart rate monitoring or subjective exertion scales (e.g., BORG scale) could enhance the assessment of mobilisation quality. The ankle-mounted device used in this study has demonstrated high accuracy in detecting steps in older adults, particularly in those with reduced gait speed [30, 31]. In addition, the four-day monitoring period appears sufficient to obtain reliable estimates of habitual activity in this population [32–34].

The implementation of a new intervention in clinical practice encounters challenges, including lack of teamwork, knowledge gaps, low motivation, limited resources and organizational rigidity [35]. This study adds to current evidence by demonstrating that a structured, theory-informed approach can support behaviour change and goal attainment under real-world clinical conditions. By combining implementation-related outcomes with objective mobility data, the findings offer practical insights into the integration of mobilisation strategies into geriatric practice and highlight areas where further support is needed.

### Limitations

Several limitations of this study should be considered when interpreting these findings. Because the two accelerometer samples comprised different inpatients, no causal inference can be drawn regarding changes in mobility. Furthermore, we did not control for weekday-related variation in step counts, which may have influenced activity levels. However, prior evidence suggests that such variability is relatively low in older adults [34]. In addition, the limited response rate – particularly the absence of physicians – constrains interpretation of profession-specific perceptions regarding acceptability, appropriateness and feasibility. This may also reduce the generalisability of the implementation findings to clinical settings with different interprofessional engagement or organisational characteristics. Moreover, the low response rate among nurses and the complete absence of physicians restrict the validity of profession-specific conclusions and may overrepresent the perspectives of the most engaged group, may contribute to an overestimation of overall acceptability and feasibility. Due to the small and heterogeneous sample, internal consistency could not be evaluated within this dataset, and perceptual implementation outcomes were analysed at the item level and as composite scores. This may limit interpretability and comparability and should be addressed in future studies with larger sample sizes. Moreover, although mobility goals were jointly defined with patients and based on therapists’ functional assessments and clinical recommendations, only nurses documented the daily objective in the MP. A more formally integrated interprofessional documentation process could strengthen shared responsibility and alignment among all HCP groups in future implementations. Although goal attainment was documented daily as part of routine clinical practice, it was not assessed using a standardized and validated measurement instrument (e.g., Goal Attainment Scaling). Satisfaction related to goal setting was not systematically assessed or documented. Consequently, neither goal attainment nor satisfaction could be analysed as separate quantitative outcomes. Furthermore, the documentation-based indicators used to assess fidelity and adoption represent pragmatic proxies of implementation behaviour rather than direct observations of intervention delivery. Consequently, fidelity and adoption estimates may over- or underestimate actual clinical practice. More comprehensive fidelity assessments should be incorporated in future research to capture the quality and consistency of MP use. Furthermore, patient perspectives were not assessed, although they are essential for a comprehensive evaluation of implementation success, particularly for interventions aiming to change patient mobility behaviour. Future studies should therefore include patient-reported experiences to support a more patient-centred assessment of acceptability and perceived benefit.

The small sample size limited the ability to draw robust conclusions regarding client outcomes. Therefore, only descriptive analyses were feasible. No power analysis was conducted prior to data collection, restricting the generalisability of the findings. Given the absence of a concurrent control group, the observed differences in daily step counts cannot be causally attributed to the Mobility Plan. Pre/post comparisons with independent inpatient samples are vulnerable to unmeasured contextual influences (e.g., variations in patient mobility potential, care processes, or organisational factors) that may have contributed to the observed changes. Moreover, the study did not assess service-level outcomes (e.g. efficiency, safety, or patient-centredness) or the satisfaction or symptomatology of the client outcomes [17, 36]. Finally, patients with moderate to severe cognitive impairment (Mini-Mental State Examination score < 20) were excluded from activity monitoring, although physical activity may be particularly beneficial for this population in preventing or mitigating neurocognitive decline [37, 38]. Across both evaluation phases, 23 inpatients were eligible for accelerometer-based mobility assessment. Some patients were not included because they met at least one exclusion criterion (prescribed bed rest, pre-existing inability to walk, insufficient German language proficiency, anticipated length of stay < 7 days, or Mini-Mental State Examination score < 20) or declined participation. While the overall proportion of included versus non-included eligible patients is known, the exact number of patients per exclusion reason was not systematically documented. This limits the transparency of the sampling process and may affect the generalisability of the mobility outcomes.

### Clinical implications

The present findings indicate that the MP is feasible and contextually appropriate to support mobility among older inpatients. The observed increase in step count and the high proportion of achieved daily goals underscore its clinical utility and relevance in practice. As this study showed, it is useful to follow a structured, theory-informed implementation approach, guided by two established frameworks and applied in a real-world geriatric care setting. This allowed for a comprehensive assessment of implementation outcomes across HCPs and contributed valuable insights into the practical integration of MPs. However, the differential levels of engagement across HCPs highlight the importance of addressing role-specific barriers and fostering shared ownership of the implementation. In particular, the limited participation of nursing staff and physicians suggests a need for tailored implementation strategies that account for profession-specific perceptions of relevance, workload implications, and role clarity. Strengthening interdisciplinary collaboration and integrating mobilisation initiatives more firmly into routine clinical workflows may enhance implementation fidelity and sustainability in geriatric care settings.

## Conclusions

The implementation of the MP yielded mixed results. While the MP appeared feasible to integrate into routine care and showed potential to support mobility among geriatric inpatients, engagement varied across HCPs. A modest increase in daily step counts was observed, although this cannot be attributed to the MP due to the study design. The structured, theory-informed implementation approach provided valuable insights into patients’ daily mobility. Future studies should include longer evaluation periods and larger samples to assess interdisciplinary uptake and examine potential effects on mobility using more robust designs.

## Supplementary Information

Supplementary Material 1.

## Data Availability

All data that are relevant for the data analysis or better understanding of this study are included in this article and its appendices.

## Abbreviations

HCP: Healthcare professional
IQR: Interquartile range
MP: Mobility Plan
SAM: StepWatch activity monitor
