# Supplementary material for: Evaluation of the implementation of a mobility plan in a geriatric clinic in Switzerland – a quality improvement study
Source: BMC Geriatr. 2026 May 23;26:936. doi: 10.1186/s12877-026-07664-8 (PMC13353024; doi:10.1186/s12877-026-07664-8)
Supplement: Supplementary file 1 — Supplementary Material 1. [file 12877_2026_7664_MOESM1_ESM.docx]

**Appendix 1**

*Flowchart of inpatient recruitment and measurement procedures*


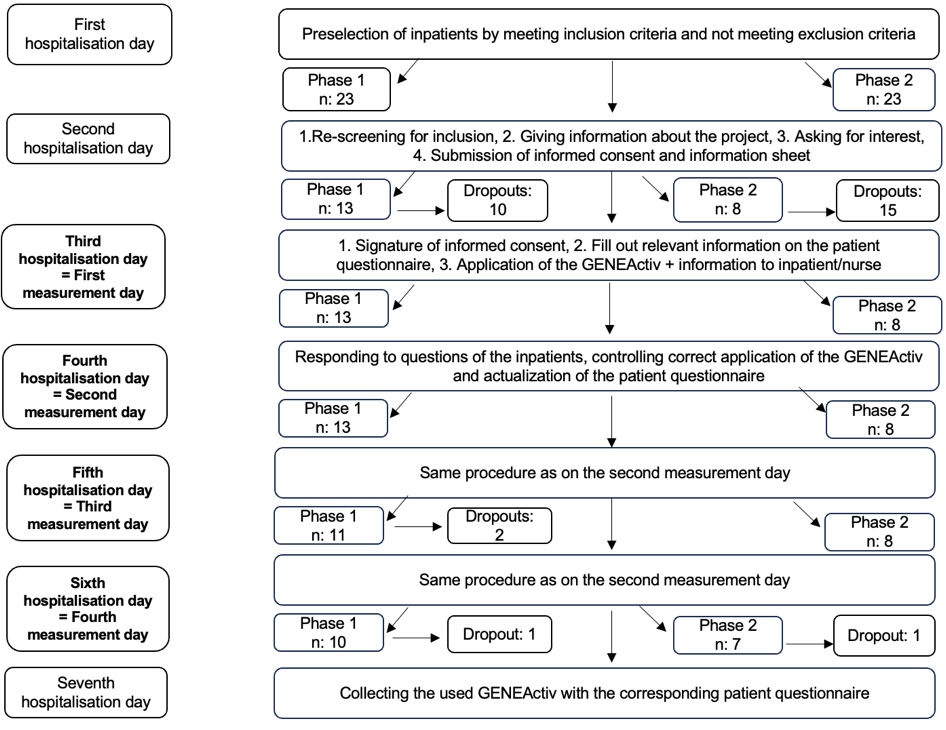


### **Appendix 2**

### *Information sheet about the use of the GENEActiv*


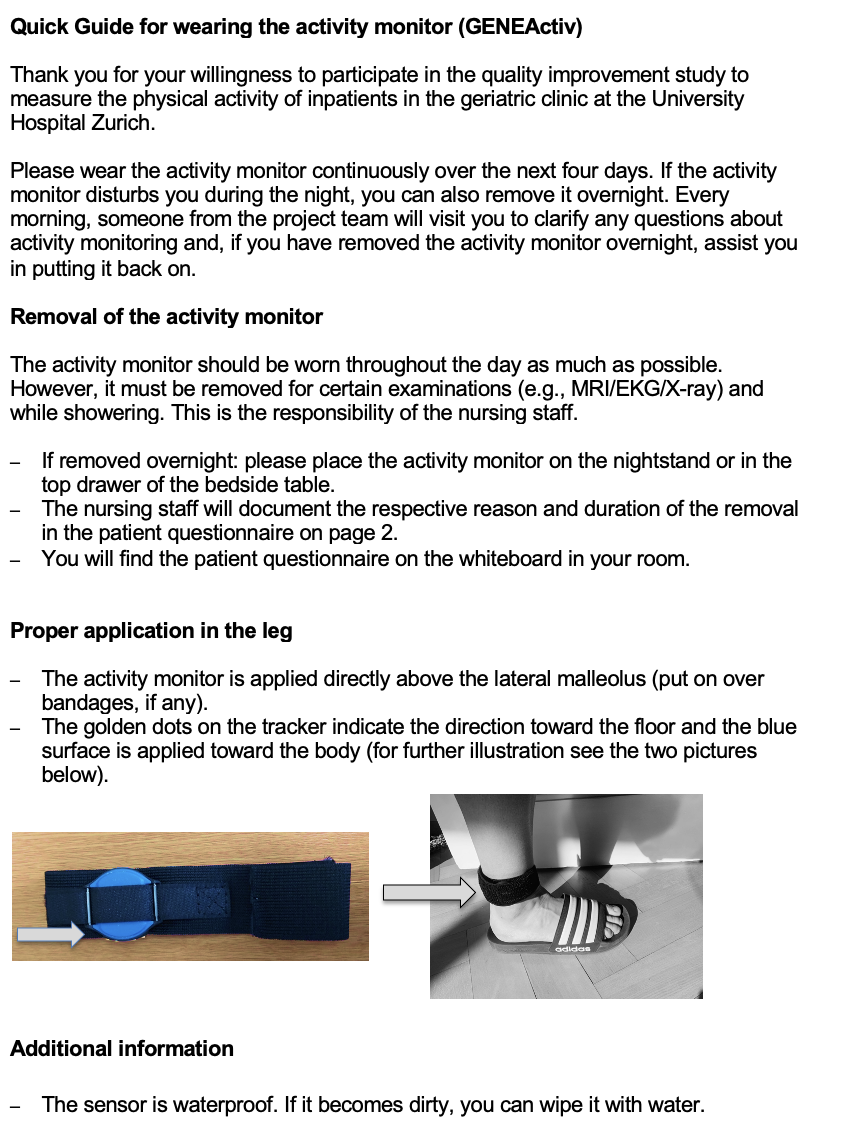


### **Appendix 3**

### *Patient questionnaire*

**
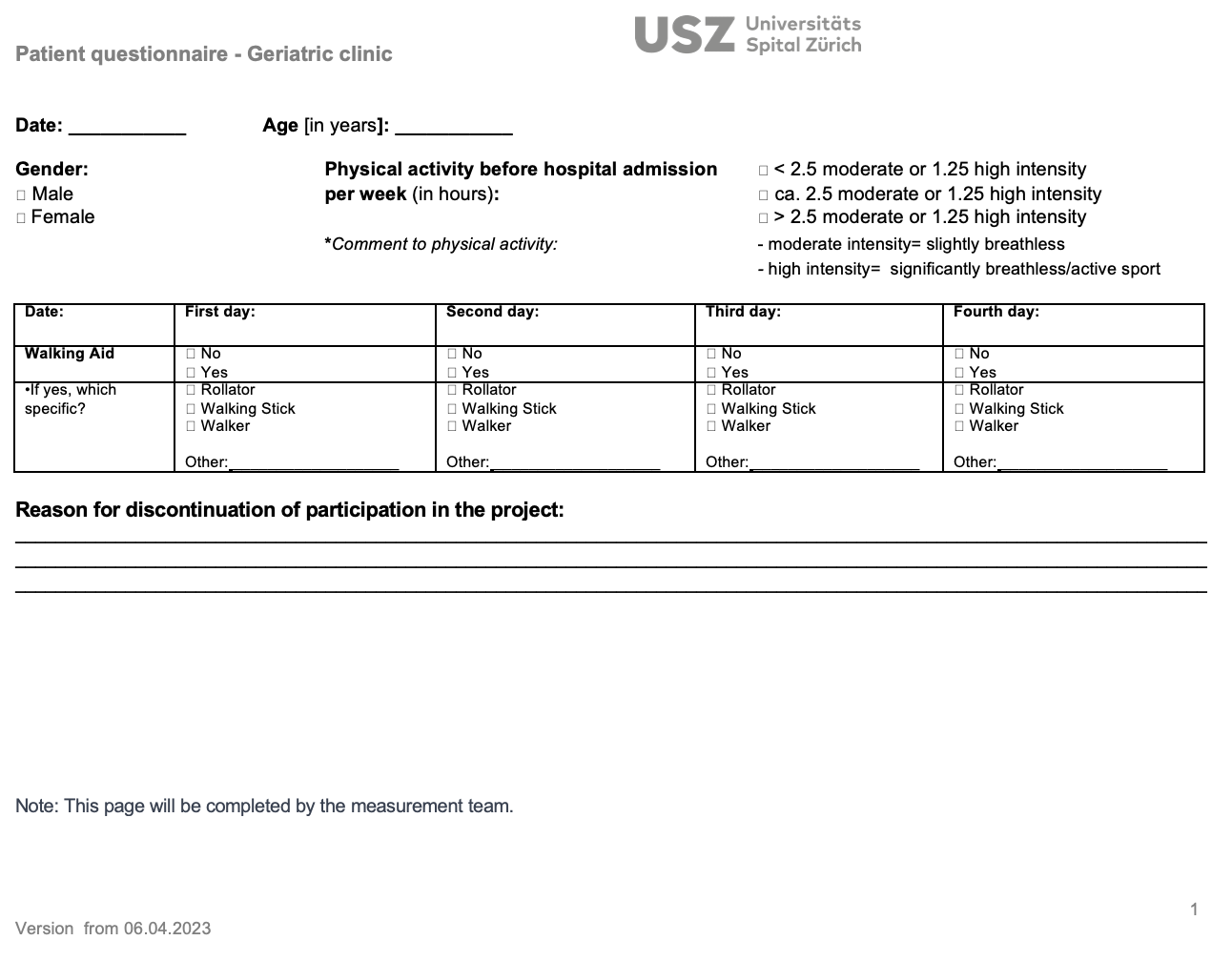
**


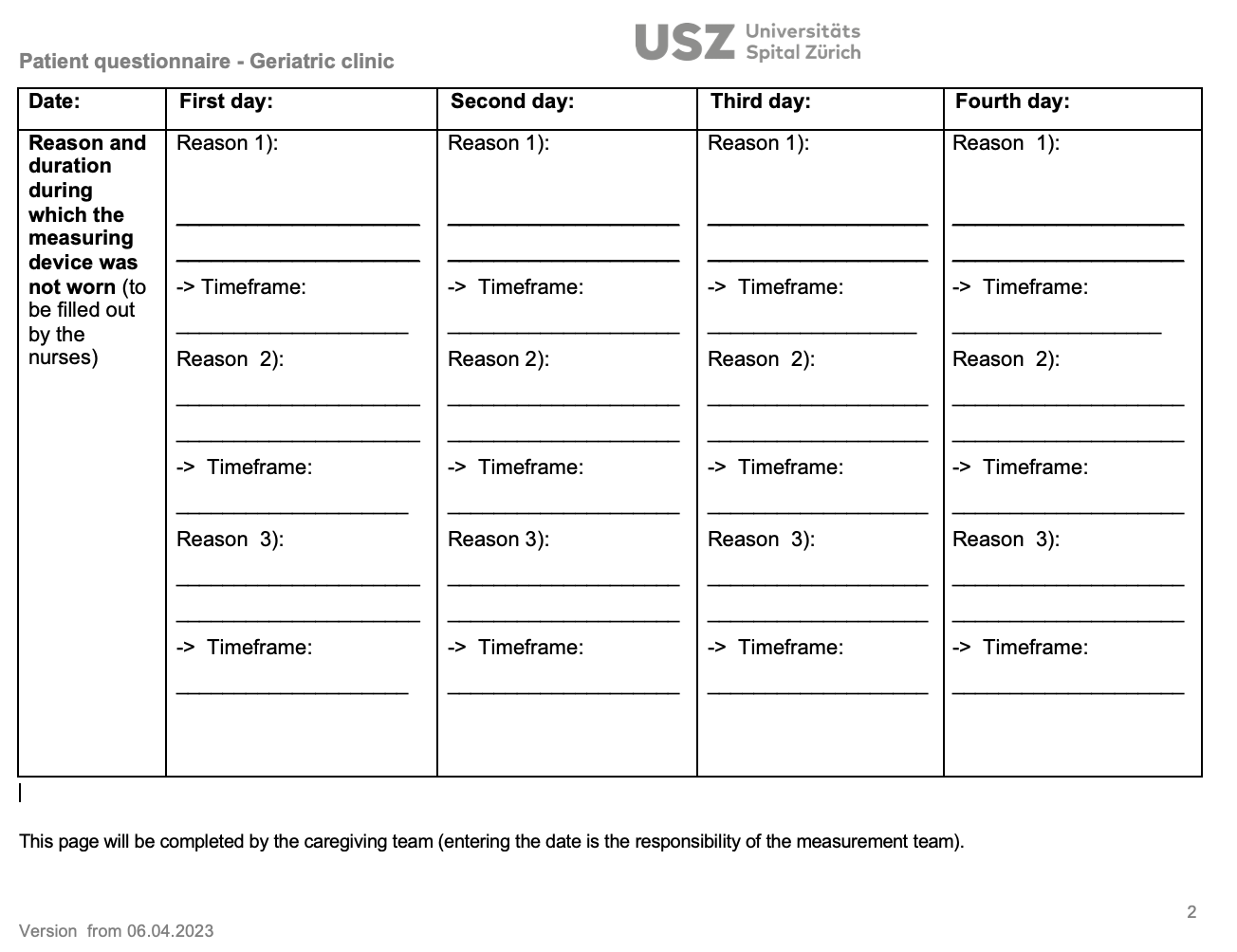


**Appendix 4**

*Final German version of the Acceptability of Intervention Measure (AIM), Intervention Appropriateness Measure (IAM), and Feasibility of Intervention Measure (FIM) ^a^*

**Allgemeine Anleitungen:** Die Skalen können einzeln oder zusammen eingesetzt werden. Die Items der *Skala zur Angemessenheit der Intervention* (IAM) können modifiziert werden, so dass sie sich auf eine spezifische Organisation, Situation oder Population (z.B.: Schüler und Schülerinnen, Patienten und Patientinnen) beziehen.

***Skala zur Akzeptabilität der Intervention (SAI)****Acceptability of Intervention Measure (AIM)*

|  | Stimme überhaupt nicht zu | Stimme nicht zu | Weder, noch | Stimme zu | Stimme vollkommen zu |
| --- | --- | --- | --- | --- | --- |
| 1. (Die Intervention X) findet meine Zustimmung. | ➀ | ➁ | ➂ | ➃ | ➄ |
| 1. (Die Intervention X) spricht mich an. | ➀ | ➁ | ➂ | ➃ | ➄ |
| 1. Ich mag (die Intervention X). | ➀ | ➁ | ➂ | ➃ | ➄ |
| 1. Ich begrüße (die Intervention X). | ➀ | ➁ | ➂ | ➃ | ➄ |

***Angemessenheit der Intervention Skala (AIS)****Intervention Appropriateness Measure (IAM)*

|  | Stimme überhaupt nicht zu | Stimme nicht zu | Weder, noch | Stimme zu | Stimme vollkommen zu |
| --- | --- | --- | --- | --- | --- |
| 1. (Die Intervention X) scheint (für A) passend zu sein. | ➀ | ➁ | ➂ | ➃ | ➄ |
| 1. (Die Intervention X) scheint (für A) geeignet zu sein. | ➀ | ➁ | ➂ | ➃ | ➄ |
| 1. (Die Intervention X) scheint (für A) brauchbar zu sein. | ➀ | ➁ | ➂ | ➃ | ➄ |
| 1. (Die Intervention X) scheint (für A) eine gute Wahl zu sein. | ➀ | ➁ | ➂ | ➃ | ➄ |

***Skala zur Machbarkeit der Intervention (SMI)****Feasibility of Intervention Measure (FIM)*

|  | Stimme überhaupt nicht zu | Stimme nicht zu | Weder, noch | Stimme zu | Stimme vollkommen zu |
| --- | --- | --- | --- | --- | --- |
| 1. (Die Intervention X) scheint umsetzbar zu sein. | ➀ | ➁ | ➂ | ➃ | ➄ |
| 1. (Die Intervention X) scheint möglich zu sein. | ➀ | ➁ | ➂ | ➃ | ➄ |
| 1. (Die Intervention X) scheint machbar zu sein. | ➀ | ➁ | ➂ | ➃ | ➄ |
| 1. (Die Intervention X) scheint benutzerfreundlich zu sein. | ➀ | ➁ | ➂ | ➃ | ➄ |

**Source English Version:** Additional File 3 in DOI: 10.1186/s13012-017-0635-3 (Weiner et al., 2017) <https://implementationscience.biomedcentral.com/articles/10.1186/s13012-017-0635-3#Sec27>

*^a^ This German version of the AIM, IAM, and FIM was retrieved from the supplementary material of Kien et al. (2021).*
